# Efficacy of Long‐Term Remote Ischemic Conditioning on Vascular and Neuronal Function in Type 2 Diabetes Patients With Peripheral Arterial Disease

**DOI:** 10.1161/JAHA.118.011779

**Published:** 2019-06-25

**Authors:** Christian S. Hansen, Marit E. Jørgensen, Jesper Fleischer, Hans Erik Bøtker, Peter Rossing

**Affiliations:** ^1^ Steno Diabetes Center Copenhagen Gentofte Denmark; ^2^ National Institute of Public Health Southern Denmark University Copenhagen Denmark; ^3^ Clinical Institute of Medicine Aarhus University Aarhus Denmark; ^4^ Department of Cardiology Aarhus University Hospital Aarhus N Denmark; ^5^ Department of Clinical Medicine University of Copenhagen Denmark

**Keywords:** diabetes mellitus, nerve function, nervous system, peripheral vascular disease, remote ischemic conditioning, vascular function, Treatment, Peripheral Vascular Disease, Vascular Disease

## Abstract

**Background:**

Peripheral artery disease is a major socioeconomic challenge in the diabetes mellitus community and non‐surgical treatment options are limited. As remote ischemic conditioning (RIC) improves vascular function and attenuates ischemia‐induced tissue damage, we investigated the efficacy of RIC on vascular and neuronal function in type 2 diabetes mellitus patients with peripheral artery disease.

**Methods and Results:**

We enrolled 36 type 2 diabetes mellitus patients with moderately reduced toe pressure (40–70 mm Hg) in a randomized sham‐controlled double‐masked trial. Patients were allocated to 12 weeks once daily upper arm cuff‐based treatment of either RIC treatment (4 cycles of 5‐minute ischemia followed by 5‐minute reperfusion) or similar sham‐device treatment. Primary outcome was transcutaneous tissue oxygen tension of the instep of the feet. Secondary outcomes were aortic pulse wave velocity, toe pressure and toe‐brachial index. Tertiary outcomes were markers of peripheral and autonomic nerve function. We enrolled 36 patients (83% men). Patients had a mean (SD) age of 70.7 years (6.8), diabetes mellitus duration of 18.4 years (8.3), HbA1c (gycated hemoglobin) of 59.7 mmol/mol (11.2). Eighty percent had peripheral symmetrical neuropathy. The mean difference in change of transcutaneous tissue oxygen tension from baseline between the RIC and sham‐treated groups was −0.03 mm Hg ([95% CI −0.1; 0.04], *P*=0.438). RIC did not elicit any change in additional outcomes. Three patients experienced transient skin petechiae in the treated arm.

**Conclusions:**

Long‐term repeated remote ischemic conditioning treatment have no effect on tissue oxygenation, vascular or neuronal function in patients with type 2 diabetes mellitus and moderate peripheral artery disease.

**Clinical Trial Registration:**

URL: http://www.ClinicalTrials.gov. Unique identifier: NCT02749942.

**Clinical Perspective**

**What Is New?**

- Peripheral artery disease is a major socioeconomic challenge in the diabetes mellitus community where remote ischemic condition may serve as a new treatment modality for peripheral artery disease.
- Thirty‐six type 2 diabetes mellitus patients with moderately reduced toe pressure were enrolled in a randomized placebo‐controlled double‐masked trial, investigating the effect of 12 weeks once daily upper arm cuff‐based treatment of either remote ischemic condition treatment (4 cycles of 5‐minute ischemia followed by 5‐minute reperfusion) or similar sham device treatment on vascular and neuronal outcomes.
- Long‐term repeated remote ischemic conditioning treatment had no effect on tissue oxygenation, vascular or neuronal function in patients with type 2 diabetes mellitus and moderate peripheral artery disease.

**What Are the Clinical Implications?**

- We show that home‐treatment is feasible and safe.
- Remote ischemic conditioning may, however, not be efficacious in patients with diabetes mellitus and neuropathy.

Peripheral artery disease (PAD) is a major challenge in the diabetes mellitus community. The prevalence of PAD in patients with diabetes mellitus exceeds that of non‐diabetic individuals and is estimated to be ≈26% in individuals aged >65 years progressing to 71% in the age range >70 years.^1^ PAD is the major cause of foot ulcers and lower extremity amputations. PAD has important socioeconomic implications and is considered the most expensive complication to diabetes mellitus.^2^ The main treatment option for PAD is surgical and endovascular revascularisation,^3^ which is associated with increased rates of complications and mortality in diabetes mellitus patients.^4^ Although pharmacological treatment with anti‐platelet drugs in patients with PAD has shown beneficial effect on walking distance,^5^ they have not been endorsed as standard treatment for PAD. No other evidence‐based treatment options are currently available. Hence, new treatment options are urgently needed for individuals with diabetes mellitus and PAD.

Remote ischemic conditioning (RIC) is a non‐invasive non‐pharmacological treatment that attenuates tissue damage caused by ischemia‐reperfusion injury. RIC has been shown capable of reducing infarct size in patients with acute myocardial infarction,^6^ reducing organ damage in patients undergoing kidney transplantation^7^ and to have neuroprotective effects in patients with stroke.^8^ RIC is achieved by brief repetitive periods of ischemia induced in an extremity eg, an arm. It is believed that the effect of RIC is mediated through both neuronal and humoral pathways. In addition to the effect on ischemia‐reperfusion injury, RIC has been shown to have beneficial effects by attenuating platelet activation and aggregation,^9^ improving endothelial function,^10^ and improving microcirculation.^11^

Only few studies have investigated the efficacy of long‐term RIC treatment. These studies have demonstrated that home‐based long‐term treatment is feasible and that it could improve endothelial function and microcirculation in healthy individuals, reduce the recurrence of stroke in patients with prior stroke^12^ and increase muscle strength and decrease blood pressure in patients with chronic heart failure.^13^ No studies have yet investigated the effect of long‐term RIC in diabetes mellitus patients. The acute effect of a single RIC treatment on walking distance in non‐diabetic patients with claudication^14^ has demonstrated a trend towards improvement. However, the results were inconclusive because of small sample sizes. We hypothesized that RIC treatment for an extended period has beneficial effects on the predominant pathophysiological components, vascular and neuronal damage, underlying PAD^2^ by attenuating the pathophysiological processes in the micro‐ and macro‐vasculature related to PAD.

The aim of the present study was to investigate the efficacy and safety of 12 weeks of RIC treatment once daily in patients with type 2 diabetes mellitus and moderate PAD.

## Methods

The data that support the findings of this study are available from the corresponding author upon reasonable request.

### Study Design

The study was a single‐center randomized double‐masked sham‐controlled trial performed at Steno Diabetes Center Copenhagen (Gentofte, Denmark). Inclusion criteria were: (1) type 2 diabetes mellitus, (2) aged 40 to 80 years, (3) and toe pressure between 40‐ and 70‐mm Hg. Exclusion criteria were: (1) active foot ulcer, (2) peripheral gangrene or infection, (3) heart failure: NYHA (New York Heart Association) class III and IV, (4) pregnancy, (5) cancer, or (6) treatment for chronic obstructive pulmonary disease. Patients were recruited from the outpatient clinic of Steno Diabetes Center Copenhagen. Patients were identified from the patient database at Steno Diabetes Center Copenhagen. Patients with confirmed symptoms of claudication and/or toe pressures between 40 and 70 mm Hg were contacted by mail or telephone and invited to the first study visit, where final eligibility was assessed by interview and toe pressure assessment. All patients gave written informed consent. The study conformed to the Declaration of Helsinki and the study protocol was approved by the local Danish ethics committee (ID H‐15019400) and the data protection agency (SDC 2015‐47) and registered at ClinicalTrials.gov (ID NCT02749942).

### Intervention

Patients were randomized to 12‐week once daily self‐administered cuff‐based treatments of either 4 cycles of 5‐minute forearm ischemia/reperfusion with a cuff pressure of 200 mm Hg or 4 cycles of 5‐minute sham device treatment with a cuff pressure of 0 mm Hg. Patients did not receive treatment on the day examination.

At the initial pre‐study visit, patients were screened for eligibility by an interview and toe pressure measurement. Eligible patients were randomized as stated below. Randomized patients were subjected to 5 subsequent study visits: baseline (randomization), after 1, 4, and 12 weeks of treatment and 4‐week post‐treatment. All outcomes measures were collected at baseline, after 4 and 12 weeks of treatment and 4 weeks post‐treatment. Toe pressure measurements were only performed at screening and end of treatment (week 12). At study visit one (week after treatment initiation) all measures besides toe pressure, arterial stiffness and cardiovascular reflex tests (CARTs) were collected.

### Randomization

Patients were randomly assigned (1:1) in blocks of 4 to receive either active cuff‐based RIC treatment by an automated RIC device (the reusable fully automated RIC device “AutoRIC”, CellAegis Devices, Canada) or cuff‐based sham treatment by a similar automated sham RIC device. A third‐party researcher not affiliated with the trial at Steno Diabetes Center Copenhagen constructed a computer‐generated randomization list. To ensure the double‐masked design, devices were packed in boxes and consecutively allocated to trial patients in concordance with the randomization list by a third‐party researcher not affiliated with the study. The personnel responsible for randomization and devices had no further involvement in the trial. Active and sham devices were indistinguishable from each other and yielded identical sounds by use. Study staff was masked to treatment allocations. During the trial patients were asked to report severe discomfort to treatment. To prevent unmasking, trial staff did not ask about other discomfort.

### Outcomes

The primary outcome of the trial was change in transcutaneous tissue oxygen tension (TcPO_2_) of the dorsal part of the foot from baseline to 12 weeks of treatment measured by the Periflux 6000 system (Perimed, Sweden). The primary end point was chosen to be tissue oxigination as this was assessed to be the most direct measures of vascularization in the end‐organ tissue. Adhesive TcPO_2_ electrode were attached to the skin 3.5 cm proximately from the root of the third toe on both feet. The position of the electrode was photographed to ensure identical electrode position at the following study visits. Probes were heated to 44°C and TcPO_2_ levels were recorded as a mean of oxygen levels from 15 to 16 minutes when plateau was reached.

#### Secondary outcome variables

Aortic (carotid–femoral) pulse wave velocity (PWV) was measured using SphygmoCor (AtCor Medical, Sydney, Australia). PWV was calculated as the time delay between carotid and femoral pulsation divided by the distance between the carotid and femoral arteries multiplied by 0.8.^15^ Three PWV measurements were recorded and averaged. Systolic blood pressure in the first toe on each foot was measured by strain gauge technique by the Digimatic DM2000 device (Medimatic, Hellerup, Denmark). Feet were preheated for 10 minutes by an electric heat blanket. Toe/brachial index was calculated by dividing the mean of 2 systolic toe pressure measurements with the mean of 2 systolic blood pressure measurements. PWV was chosen as a secondary outcome because previous studies affecting risk factors for reduced PWV such as inflammation^16^ has been demonstrated to be improved by RIC treatment.^17^ Toe pressure was chosen as an outcome because potential risk factors for reduced toe pressure such as inflammation and increased platelet activation and aggregation and reduced endothelial function was been shown to be improved be RIC treatment.^8^, ^10^ Both outcomes are established markers of cardiovascular disease.

#### Tertiary outcome variables

Trained technicians used a Vagus device (Medicus Engineering, Aarhus, Denmark) to quantify cardiovascular autonomic neuropathy. Indices of 5‐minute supine resting heart rate variability and the 3 standard cardiac autonomic reflex tests (CARTs) recommended for diagnosing cardiovascular autonomic neuropathy^18^ were performed: the lying‐to‐standing test (30/15), the deep breathing test (E/I ratio) and the Valsalva maneuver. CARTs were performed in the mentioned order and in accordance with procedures as described previously.^19^ CARTs and heart rate variability measures were analyzed as continuous variables. We used age‐dependent cut‐off levels^18^ to define pathological results of the CARTs. The cardiovascular autonomic neuropathy diagnosis was defined as the presence of 2 or 3 pathological CARTs.

Peripheral small‐fiber autonomic function was assessed by electrochemical skin conduction test on the hands and feet by the Sudoscan device (Impeto Medical, Paris, France). Age and sex stratified electrochemical skin conduction thresholds for hands and feet were used.^20^ Sural nerve conduction velocity and sural nerve action potential (SNAP) were measured using the handheld NC‐Stat *DPNCheck* (NeuroMetrix, Inc, Waltham, USA). Age and height stratified threshold limits for sural nerve action potential and sural nerve conduction velocity were applied to identify abnormal results.^21^

Vibration perception threshold was determined using a Bio‐Thesiometer (Bio‐Medical Instruments, OH, USA) at the distal end of the great toe on both feet. Age stratified perception thresholds were used to determine pathological vibration perception threshold.^22^ Light touch perception was assessed by applying a 10‐g monofilament to 3 points at the footpads just proximal to the first, third, and fifth toe. Pain sensation assessment was performed using a 40‐g pin‐prick device (Neuropen; Owen Mumford Ltd, Oxford, UK) applied at the dorsal side of the first, third, and fifth toe just proximal to the nail on both feet. We applied Neuropen assessments 3 times at each point and confirmed sensation only when the patient indicated sensation at all 3 stimuli. Symptoms of peripheral neuropathy were assessment by a composite score of the Minnesota Neuropathy Screening Instrument (MNSI) questionnaire. A score >6 was defined as peripheral neuropathy.

All examinations were performed in a quiet setting at room temperature (18°C –23°C) between 8 am and 12 am. Patients started fasting at midnight before testing, refrained from smoking on the day of examination, and avoided strenuous exercise 24 hours before examination. Patients did not take any medication on examination days.

Neuropathy outcomes were chosen as exploratory outcomes because diabetic neuropathy is a serious complication to diabetes mellitus which could be affected by RIC treatment, as RIC has been demonstrated to improve measures of microcirculation^11^ which could affect nerve function directly. Also, a RIC‐induced reduction in systemic inflammation (as mentioned above) could improve nerve function.

### Anthropometric Variables

Height and weight were measured with light indoor clothing, without shoes, using a fixed rigid stadiometer (Seca, Chino, USA) and an electronic scale (Mettler Toledo, Glostrup, Denmark), respectively.

### Blood Pressure

Oscillometric (A&D Medical, UA787) office blood pressure was measured in a supine position after 15 minutes rest using an appropriate cuff size. Three measurements were obtained and averaged.

### Biochemical Variables

HbA1c was analyzed by high‐performance liquid chromatography on a Tosoh G7 (Tosoh Corporation, Japan). High density lipoprotein and total cholesterol were analyzed by standard enzymatic colorimetry techniques. Creatinine was analyzed by 2‐point rate enzymatic technique. Urinary albumin excretion ratio was measured in morning spot urine collections by an enzyme immunoassay. Urinary albumin was analyzed by quantitative immunological turbidimetry.

All analyses except for HbA1c were done on a Vitros 5600 (Orhto Clinical Diagnostics, France). Chronic Kidney Disease Epidemiology Collaboration Equation was used to calculate the estimated glomerular filtration rate from p‐creatinine.

### Lifestyle Variables

Lifestyle measures were obtained by questionnaires. Patients were classified as current smokers when using ≥1 cigarettes or cigars or pipes per day. Physical activity was defined as being regularly physically active or not.

### Compliance

Compliance was monitored at every study visit by obtaining the number of treatments through a compliance monitoring system embedded in the cuffs and read by a wireless monitor unit.

### Statistical Analyses

#### Power calculation

We hypothesized that RIC treatment would increase tissue oxygen tension in the feet (primary outcome) by 13% (SD=10%), which is an ≈30% smaller effect than previously demonstrated in healthy volunteers.^23^ With 90% power and a 2‐sided significance level of 0.05 the sample size needed to detect this change is 14 in each group or 28 patients in total. Expecting a dropout rate of 10% the sample size of the study was calculated to be 16 patients in each group.

Patient characteristics are presented as means (SD), as medians with interquartile ranges or as percentages. Group differences in baseline variables were assessed by *t* test and Chi‐square test for categorical variables. Associations were modeled by linear mixed‐effect models with a patient‐specific random intercept to account for the correlation of repeated measurements within patients using the proc GLIMMIX procedure with “variance components” as covariance structure. All analyses were performed as an intention‐to‐treat analysis. To fulfill the requirement of a normal distribution of the model residuals, outcomes were log‐transformed when applicable. Consequently, estimates for these models are given in percentages. Heart rate variability indices were adjusted for 5‐minute resting heart rate at the time of testing and PWV measures were adjusted for mean arterial blood pressure. Additional exploratory adjustments were done for relevant baseline confounders where significant or near‐significant between group differences were found. Statistical significance was inferred at a 2‐tailed *P*<0.05. All analyses including the power calculation were performed using SAS version 9.4 (SAS Institute, Cary, NC).

## Results

**Figure 1:**
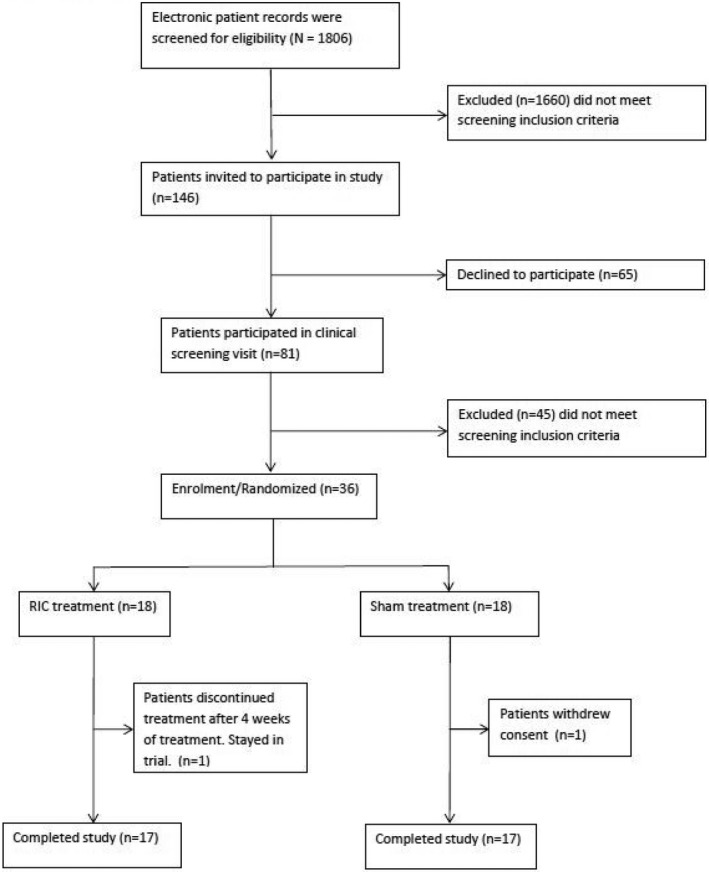
Trial profile. RIC indicates remote ischemic conditioning.

We tested 1806 patients at the initial screening of patient records. A total of 1660 did not meet the screening inclusion criteria. We invited 146 patients, of whom 65 declined the invitation. We screened 81 patients at the first study visit. Of these, 45 did not meet inclusion criteria because toe pressure measurements were beyond the inclusion range. A total of 36 patients were enrolled in the study and randomized to either active treatment or sham in equal proportions. We allocated 18 patients to active treatment and 18 to sham. One patient in the active arm discontinued treatment because of side effects but stayed in the study. One patient in the sham group withdrew consent before the second study visit, thus 34 patients completed the study (Figure 1).

Baseline data for the RIC and the sham group are shown in Table 1. Overall, 83% of patients were men, had a mean (SD) age of 70.7 years (6.8), a diabetes mellitus duration of 18.4 years (8.3), a toe pressure of 61.3 mm Hg (15.1), a TcPO_2_ for both feet of 51.3 mm Hg (10.7), an HbA1c of 59.7 mmol/mol (11.2), and 24 patients (80%) had peripheral symmetrical neuropathy assessed by vibration detection threshold. The patients did not differ between the 2 groups for any demographic, anthropometric, cardiometabolic, or neuropathic variables except for sural nerve conduction velocity (Tables 1 and 2).

**Table 1:** Baseline Characteristics

	RIC Treatment (n=18)	Sham (n=17)	*P* for Group Difference
Sex (male), n/%	14/77.8	15/88.2	0.407
Age, y	71.1 (5.5)	70.2 (8.1)	0.697
HbA1c, mmol/mol	56.9 (7.8)	62.6 (13.6)	0.110
HbA1c (%)	7.4 (0.7)	7.9 (1.2)	0.110
Body mass index, kg/m^2^	32.0 (5.3)	30.8 (4.9)	0.465
Weight, kilo	93.0 (17.6)	90.9 (10.2)	0.664
Current smoker, n/%	3/16.7	5/29.4	0.364
No exercise, n/%	6/33.3	6/35.3	0.903
Systolic blood pressure, mm Hg	145.7 (11.4)	151.9 (17.5)	0.202
Diastolic blood pressure, mm Hg	76 (7.8)	76.1 (8.2)	0.982
Diabetes mellitus duration, y	19.2 (6.4)	17.5 (9.9)	0.525
Myocardial infarction, self‐reported, n/%	3/16.7	5/29.4	0.364
Cerebral infarction, self‐reported, n/%	3/16.7	5/29.4	0.364
Thrombosis in leg, self‐reported, n/%	0/0	3/17.6	n/a
Impaired sural nerve conduction, n/%^a^	13/100	8/61.5	0.004
Impaired sudomotor function feet, bilateral, n/%	13/72.2	13/81.3	0.533
Impaired sudomotor function hands, bilateral, n/%	7/38.9	8/50	0.512
Impaired vibration sensation, bilateral, n/%	9/69.2	15/88.2	0.185
Monofilament sensation (total of 8, both feet)	4.7 (3.6)	6.1 (3.1)	0.209
Pain (Pin prick) sensation (total of 6, both feet)	3.7 (2.4)	4.1 (2.1)	0.642
Peripheral neuropathy (MNSI count >6), n/%	7/41.2	1/9.1	0.050
Cardiovascular autonomic neuropathy diagnosis, n/%	4/26.7	0/0	n/a
Claudication (Edinburgh claudication questionnaire)	3/16.7	4/23.5	0.611
Mean great toe pressure, mm Hg	61.4 (15.7)	61.2 (15.0)	0.967
Mean toe brachial index	0.4 (0.3–0.5)	0.4 (0.3–0.5)	0.744
eGFR, mL/min per 1.73 m^2^	55.0 (40.7–73.5)	61.9 (52.5–68.5)	0.668
Urinary albumin excretion rate (mg/24‐h)	27 (14–195)	57 (22–285)	0.393
Total cholesterol, mmol/L	3.4 (3.1–4.4)	4.0 (3.5–4.7)	0.112
HDL cholesterol, mmol/L	1.1 (0.3)	1.1 (0.1)	0.540
LDL cholesterol, mmol/L	1.7 (1.2–2.2)	2.1 (1.4–2.8)	0.228
Triglycerides cholesterol, mmol/L	1.6 (1–2.6)	2.0 (1.2–3.2)	0.194
*Medication*			
Beta blocker, n/%	10/55.6	7/41.2	0.390
Diuretic, n/%	5/27.8	5/29.4	0.915
RAAS blocker, n/%	7/38.9	6/35.3	0.826
Statins, n/%	15/83.3	12/70.6	0.364
Calcium antagonists, n/%	8/44.4	7/41.2	0.845
Any lipid lowering, n/%	16/88.9	15/88.2	0.952
Glp1‐receptor agonist, n/%	8/44.4	4/23.5	0.182
DPP4 inhibitor, n/%	2/11.1	6/35.3	0.075
SGLT‐2 inhibitor, n/%	1/5.6	5/29.4	0.048
Metformin, n/%	14/77.8	12/70.6	0.626
Long‐acting insulin, n/%	10/55.6	9/52.9	0.877
Intermediate‐acting insulin, n/%	1/5.6	3/17.6	0.252
Fast‐acting insulin, n/%	11/61.1	8/47.1	0.400
Vitamin K antagonists, n/%	2/11.1	1/5.9	0.579
Acetylsalicylic acid, n/%	2/11.1	3/17.6	0.579
Antiplatelet drugs, n/%	4/22.2	6/35.3	0.387

Data are in means (SD), medians (interquartile ranges) or n (%). eGFR indicates estimated glomerular filtration rate (mL/min per 1.73 m^2^); HDL, high‐density lipoprotein; LDL, low‐density lipoprotein; MNSI, Michigan Neuropathy Screening Instrument questionnaire; RAAS, renin angiotensin aldosterone system; HbA1c, Glycated Heemoglobin; Glp 1, Glucagon Like Peptide 1; DPP4, dipeptidyl peptidase 4; SGLT‐2, Sodium‐glucose co‐transporter 2.

a Where measures were achievable.

**Table 2:** Baseline Characteristics for Outcome Variables

	Randomization		
	Active Treatment	Sham Treatment	*P* Value for Group Difference
Primary outcome			
Transcutaneous oxygen tension, right foot (mm Hg)	50.6 (10.1)	49.0 (6.8)	0.580
Transcutaneous oxygen tension, left foot (mm Hg)	50.9 (11.4)	52.3 (13.5)	0.732
Transcutaneous oxygen tension, mean (mm Hg)	50.8 (9.9)	51.8 (11.8)	0.763
Secondary outcomes			
Pulse wave velocity, m/s	14.0 (3.1)	13.3 (3.4)	0.487
Toe pressure, mean (mm Hg)	61.4 (15.7)	61.2 (15.0)	0.967
Toe brachial index, mean	0.41 (0.31–0.48)	0.36 (0.33–0.45)	0.684
Tertiary outcomes			
E/I ratio	1.06 (1.04–1.23)	1.11 (1.09–1.2)	0.929
30/15 ratio	1.06 (1.02–1.13)	1.06 (1.03–1.11)	0.810
Valsalva	1.20 (1.13–1.25)	1.22 (1.1–1.43)	0.607
SDNN, ms	18.85 (11.3–38.9)	28.7 (20.8–50.5)	0.219
RMSSD, ms	13.55 (7.05–26.00)	21.6 (10.8–65.9)	0.217
Total power, ms^2^	156.89 (43.68–551.86)	294.49 (91.86–920.03)	0.223
High frequency power, ms^2^	38.83 (10.51–226.18)	59.86 (35.62–398.43)	0.336
Low frequency power, ms^2^	16.06 (6.42–100.05)	38.52 (11.23–246.69)	0.357
LF/HF ratio	1.75 (1.04–3.23)	1.67 (1.30–3.88)	0.802
Electrochemical skin conduction, hands mean (μS)	57.8 (36.5–65.8)	46.0 (38.6–61.1)	0.592
Electrochemical skin conduction, feet mean (μS)	58.9 (27.8–71.8)	54.1 (41.4–64.4)	0.225
Sural nerve conduction velocity, mean (m/s)	35.0 (5.3)	40.4.(5.7)	0.007
Sural nerve amplitude potential, mean (μV)	3.2 (2.3–4.0)	3.1 (2.7–4.4)	0.554
Vibration sensation threshold, mean (V)	24.3 (13.8–33.0)	36.5 (25.3–42.6)	0.050
Monofilament, mean both feet (number of positive responses)	6 (1–8)	8 (5–8)	0.642
Pin prick, mean both feet (number of positive responses)	4 (2–6)	5 (3–6)	0.544
Michigan neuropathy screening instrument (count)	5.7 (2.6)	3.8 (2.6)	0.058

Data are means (SD) or medians (interquartile ranges). 30/15 ratio indicates heart rate response to standing; E/I ratio, heart rate response to deep breathing; HF, high‐frequency; LF, low‐frequency; RMSSD, the root mean square of the sum of the squares of differences between consecutive R–R intervals; SDNN, standard deviation of normal‐to‐normal intervals.

Patients in both groups had high compliance to treatment applying treatment in 92.1% (SD 13.8) and 82.1% (SD 18.1) of possible days in the RIC group and the sham group, respectively, with a borderline significant group difference *P*=0.059.

**Figure 2:**
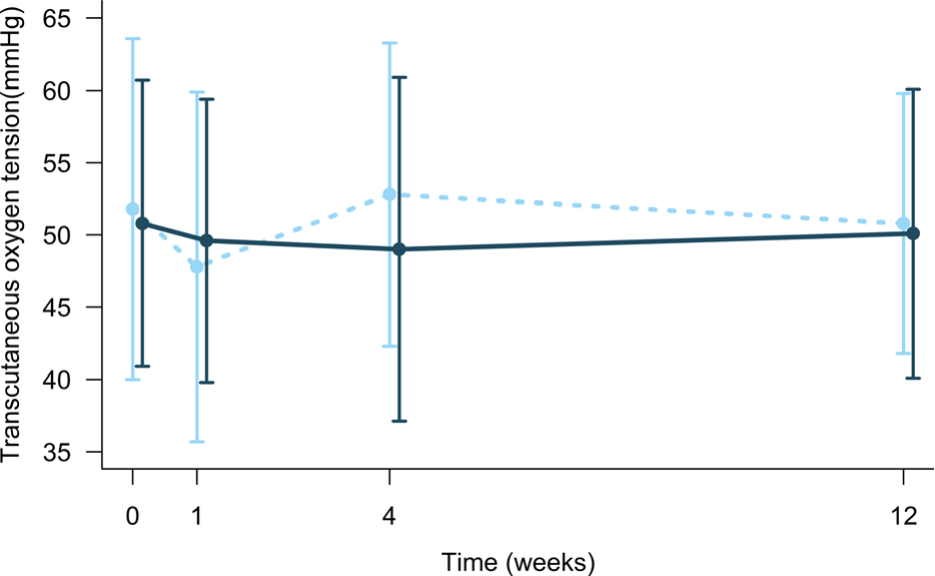
Treatment effect on transcutaneous oxygen tension. Effect of treatment (dark blue solid lines) vs sham (light blue dashed lines) during trial. Data are in mean (SD). No *P*<0.05 for group difference at any time point.

RIC did not improve the primary end point significantly, TcPO_2_ on the dorsal part of the foot, at 12 weeks either when mean values of both feet were assessed (Figure 2) nor when each foot was assessed. Mean difference in change in average TcPO_2_ from baseline to 12 weeks between groups was −0.03 mm Hg (95% CI −0.1; 0.04) by assessment on both feet (*P*=0.438 [Table 3]). No effect of RIC treatment on TcPO_2_ was observed at any other visit during treatment or at 4 weeks post‐treatment (Table 4).

**Table 3:** Effect for Trial

	Week 1			Week 4			Week 12		
	Active Treatment	Sham	Active vs Sham Difference in Change From Baseline	Active Treatment	Sham	Active vs ShamDifference in Change From Baseline	Active Treatment	Sham	Active vs Sham Difference in Change From Baseline
Primary outcome									
Transcutaneous oxygen tension, right foot (mm Hg)	49.7 (10.7)	46.4 (10.9)	0.37 (−0.39–1.12) [0.348]	46.7 (14.4)	51.9 (11.2)	0.01 (−0.23–0.25) [0.942]	48.4 (13.1)	50.1 (8)	0.00 (−0.08–0.08) [0.932]
Transcutaneous oxygen tension, left foot (mm Hg)	49.5 (11.5)	47.4 (12.7)	0.09 (−0.78–0.97) [0.836]	51.3 (10.7)	52.3 (12.7)	−0.29 (−0.53–−0.05) [0.021]	51.8 (9.7)	50.3 (10.3)	−0.07 (−0.15–0.01) [0.084]
Transcutaneous oxygen tension, mean (mm Hg)	49.6 (9.8)	47.8 (12.1)	0.31 (−0.39–1.02) [0.394]	49.0 (11.9)	52.8 (10.5)	−0.10 (−0.32–0.11) [0.353]	50.1 (10.0)	50.8 (9.0)	−0.03 (−0.1–0.04) [0.438]
Secondary outcomes									
Pulse wave velocity, m/s			n/a	13.7 (3.8)	13.2 (3.4)	−0.22% (−0.67–0.24) [0.364]	14.7 (3.6)	13.3 (3.3)	0.07% (−0.08–0.22) [0.385]
Toe pressure, mean (mm Hg)			n/a			n/a	73.3 (14.8)	75.7 (15.3)	−0.04 (−0.2–0.11) [0.603]
Toe‐brachial index, mean			n/a			n/a	0.51 (0.44–0.54)	0.49 (0.43–0.65)	−0.06% (−0.28–0.17) [0.627]
Tertiary outcomes									
E/I ratio	n/a	n/a	n/a	1.08 (1.02–1.13)	1.11 (1.07–1.15)	−0.13% (−0.42–0.15.) [0.369]	1.05 (1.04–1.14)	1.08 (1.07–1.14)	0.01% (−0.09–0.1) [0.910]
30/15 ratio	n/a	n/a	n/a	1.03 (1.01–1.06)	1.07 (1.04–1.09)	−0.12% (−0.26–0.03) [0.120]	1.04 (1.02–1.09)	1.09 (1.03–1.11)	−0.02% (−0.07–0.02) [0.296]
Valsalva	n/a	n/a	n/a	12 1.21 (1.17–1.28)	1.21 (1.09–1.25)	0.18% (−0.22–0.58) [0.402]	1.23 (1.15–1.29)	8 1.18 (1.08–1.31)	0.03% (−0.12–0.17) [0.713]
SDNN, ms	15.4 (13.9–26.9)	17.5 (14.1–42.3)	1.21% (−4.78–7.58) [0.703]	17.3 (11.6–39.2)	19.7 (16.1–38.9)	0.32% (−1.03–1.69) [0.644]	19 (10.1–28.7)	21.8 (17.6–36.9)	0.15% (−0.4–0.69) [0.604]
RMSSD, ms	12 (8.7–20)	11.8 (7.1–33.3)	2.72% (−5.09–11.17) [0.513]	9.6 (8.0–23.1)	13.0 (10.3–49.9)	0.35% (−1.3–2.02) [0.684]	11.9 (8.2–21.8)	18 (7.8–38.8)	0.14% (−0.48–0.76) [0.666]
Total power, ms^2^	98.63 (30.83–202.88)	106.37 (56.34–897.44)	1.18% (−10.98–15.00) [0.859]	135.98 (31.48–437.09)	159.06 (117.67–986.22)	1.06% (−2.37–4.61) [0.553]	138.97 (33.45–338.49)	154.56 (110.62–342.25)	0.63% (−0.71–1.99) [0.359]
High frequency power, ms^2^	35.09(5.36–50.64)	21.15 (8.22–344.23)	0.32% (−16.19–20.07) [0.973]	30.59 (8.4–122.07)	29.3 (21.36–205.19)	1.52% (−2.21–5.38) [0.434]	11.99 (8.76–78.79)	27.14 (23.29–97.64)	0.12% (−1.41–1.67) [0.877]
Low frequency power, ms^2^	14.07(5.76–52.8)	16.27 (4.77–95.62)	4.84% (−12–24.9) [0.602]	10.34 (6.38–61.77)	22.83 (10.98–227.18)	−0.60% (−3.93–2.86) [0.732]	14.52 (6.77–45.65)	22.18 (8.77–77.78)	0.07% (−1.16–1.31) [0.912]
LF/HF ratio	1.89 (0.72–2.4)	3.39 (1.21–3.6)	−4.14% (−13.38–6.08) [0.422]	1.29 (1.06–3.07)	1.50 (0.46–3.47)	2.01% (−0.14–4.2) [0.072]	1.22 (1.05–1.78)	1.25 (0.95–2.67)	0.08% (−0.72–0.88) [0.852]
Electrochemical skin conduction, hands mean (μS)	55.5 (35.8–63.8)	48.3 (43.0–63.5)	−0.68% (−2.69–1.37) [0.516]	55.3 (32–6.3)	51.3 (35.5–66.8)	−0.31% (−0.88–0.27) [0.304]	47.6 (42.4–59.0)	52.6 (40.7–60.7)	0.03% (−0.20–0.26) [0.804]
Electrochemical skin conduction, feet mean (μS)	57.3 (24.8–62.8)	54.3 (36.5–67.5)	0.25% (−2.16–2.73) [0.840]	55.8 (21.5–65.5)	46.8 (36.5–56.5)	−0.30% (−1.06–0.46) [0.438]	53.8 (30.9–68.1)	53.4 (34.5–60.3)	0.11% (−0.18–0.41) [0.445]
Sural nerve conduction velocity, mean (m/s)	42.0 (7.5)	39.7 (7.6)	2.51% (0.36–4.71) [0.030]	40.3 (3.4)	40.6 (5.1)	0.35% (−0.13–0.84) [0.157]	37.8 (4.3)	40.9 (8.1)	0.02% (−0.16–0.19) [0.861]
Sural nerve amplitude potential, mean (μV)	3.37 (3.0–4.0)	3.0 (2.8–5.5)	−0.47% (−3.74–2.92) [0.786]	3.0 (2.4–4.5.0)	3.7 (2.6–6.7)	−0.44% (−1.24–0.37) [0.295]	3.5 (2.8–4.3)	3.2 (3.0–5.8)	−0.13% (−0.38–0.13) [0.326]
Vibration sensation threshold, mean (V)	n/a	n/a	n/a	36.0 (20.0–42.0)	33.3 (24.7–40.5)	0.85% (0.05–1.65) [0.047]	31.1 (19.8–39.8)	25.3 (18.8–36.5)	0.52% (0.2–0.84) [0.003]
Monofilament, mean both feet (number of positive responses)			n/a	6.5 (1.0–8.0)	8.0 (3.0–8.0)	0.96% (−0.14–2.07) [0.100]	7.0 (0.0–8.0)	8.0 (3.0–8.0)	0.22% (−0.12–0.56) [0.218]
Pin prick, mean both feet (number of positive responses)	n/a	n/a	n/a	4.5 (2.0–6.0)	6.0 (4.0–6.0)	−0.68% (−1.35–−0.01) [0.056]	6.0 (2.0–6.0)	6.0 (4.0–6.0)	0.14% (−0.15–0.43) [0.353]
Michigan neuropathy screening instrument (count)	4.8 (2.7)	4.1 (2.6)	−0.13 (−0.3–0.04) [0.146]	5.5 (2.4)	3 (1.7)	−0.02 (−0.07–0.04) [0.581]	5.3 (2.6)	4.3 (3)	−0.01 (−0.02–0.01) [0.391]

Data are means (SD) or medians (interquartile ranges). Estimates of treatment effect are in percentages or absolute values (95% CI) [*P* values for group difference]. Models have been adjusted for baseline values of the given outcome. 30/15 ratio indicates heart rate response to standing; E/I ratio, heart rate response to deep breathing; HF, high‐frequency; LF, low‐frequency; RMSSD, the root mean square of the sum of the squares of differences between consecutive R–R intervals; SDNN, standard deviation of normal‐to‐normal intervals.

**Table 4:** Effect of Trial Post‐Treatment

	Week 4 Post‐Treatment		
	Active Treatment	Sham	Active vs Sham Difference in Change From Week 12
Primary outcome			
Transcutaneous oxygen tension, right foot (mm Hg)	51.6 (8.4)	49.8 (9.7)	0.11 (−0.09–0.31) [0.285]
Transcutaneous oxygen tension, left foot (mm Hg)	52.6 (9.1)	46.2 (14.5)	0.08 (−0.16–0.32) [0.511]
Transcutaneous oxygen tension, mean (mm Hg)	52.1 (7.7)	48.8 (11.8)	0.08 (−0.09–0.25) [0.359]
Secondary outcomes			
Pulse wave velocity, m/s	15.0 (4.5)	15.0 (4.5)	−0.11% (−0.84–0.62) [0.766]
Toe pressure, mean (mm Hg)	n/a	n/a	n/a
Toe brachial index, mean	n/a	n/a	n/a
Tertiary outcomes			
E/I ratio	1.06 (1.03–1.19)	1.06 (1.04–1.24)	−0.02% (−0.4–0.36) [0.914]
30/15 ratio	1.02 (1–1.07)	1.1 (1.02–1.13)	−0.11% (−0.25–0.02) [0.104]
Valsalva	1.2 (1.12–1.27)	1.2 (1.12–1.25)	−0.27% (−0.84–0.29) [0.359]
SDNN, ms	17.8 (8.7–49.8)	21.3 (14.7–32.6)	1.28% (−0.04–2.62) [0.07]
RMSSD, ms	15.1 (7.5–34.9)	17.3 (10.3–27.7)	1.23% (−0.59–3.07) [0.200]
Total power, ms^2^	104.65 (21.37–831.8)	155.76 (83.88–415.59)	1.86% (−1.08–4.89) [0.230]
High frequency power, ms^2^	25.42 (2.65–387.59)	48.97 (13.28–118.14)	1.84% (−2.38–6.23) [0.408]
Low frequency power, ms^2^	10.84 (5.09–123.17)	33.1 (14.63–54.83)	1.31% (−2.5–5.27) [0.514]
LF/HF ratio	1.4 (0.36–3.31)	0.97 (0.67–1.95)	0.63% (−1.35–2.64) [0.545]
Electrochemical skin conduction, hands mean (μS)	40.5 (29–6.75.0)	55.8 (36.3–61.3)	−0.40% (−1.01–0.22) [0.217]
Electrochemical skin conduction, feet mean (μS)	46.3 (27.5–63.5)	51.0 (31.8–61.3)	−0.44% (−1.08–0.2) [0.191]
Sural nerve conduction velocity, mean (m/s)	37.6 (4.3)	40.3 (7.8)	−0.04% (−0.60–0.51) [0.875]
Sural nerve amplitude potential, mean (μV)	3.7 (2.7–4.5)	3.3 (2.7–4.7)	0.83% (0.05–1.62) [0.048]
Vibration sensation threshold, mean (V)	28.5 (20.5–43)	34.8 (30.0–39.0)	−0.36% (−1.45–0.74) [0.529]
Monofilament, mean both feet (number of positive responses)	7.0 (0.0–8.0)	7.0 (4.0–8.0)	−0.11% (−0.84–0.62) [0.766]
Pin Prick, mean both feet (number of positive responses)	6.0 (2.0–6.0)	6.0 (3.0–6.0)	−0.41% (−1.62–0.82) [0.519]
Michigan neuropathy screening instrument (count)	5.1 (2.9)	5.1 (3.4)	−0.03 (−0.08–0.02) [0.197]

Data are means (SD) or medians (IQR). Estimates of treatment effect are in percentages or absolute values (95% CI) [*P* values for group difference]. Models have been adjusted for values at week 12 of the given outcome. 30/15 ratio indicates heart rate response to standing; E/I ratio, heart rate response to deep breathing; HF, high‐frequency; LF, low‐frequency; RMSSD, the root mean square of the sum of the squares of differences between consecutive R–R intervals; SDNN, standard deviation of normal‐to‐normal intervals.

Similarly, secondary outcomes were not affected by RIC at any study visit. At week 12, aortic pulse wave velocity was unaffected by RIC treatment compared with sham with a difference in change of 0.07% (95% CI −0.08; 0.22), (*P*=0.385) The difference in mean toe pressure for both toes was −0.04 mm Hg (95% CI −0.2; 0.11) (*P*=0.603). For mean toe/brachial index no differences were observed. Group difference was −0.06% (95% CI −0.28; 0.17) (*P*=0.627) (Tables 3 and 4).

Tertiary outcome variables were unaffected by RIC. Central autonomic variables assessed by cardiovascular autonomic reflex tests and indices of heart rate variability were unaffected as were peripheral autonomic variables assessed by electrochemical skin conductance. Peripheral nerve function was not affected by RIC when assessed by sural nerve conduction velocity and action potential, vibration sensation threshold and light touch sensation or when small fiber nerve function was assessed by 40‐g pin‐prick induced pain (Tables 3 and 4). RIC did not elicit significant changes in the mean score of the MNSI questionnaire (Tables 3 and 4).

As a significant between‐group difference in mean sural nerve conductance velocity was seen at baseline (Table 2), models for vascular outcomes were additionally adjusted for this possible confounder for patients with baseline measure of the confounder. A treatment effect was seen as a reduction in transcutaneous oxygen tension on the right foot at week 4 and week 12 of 0.36 mm Hg (95% CI −0.59; −0.13) (*P*=0.003) and 0.09 mm Hg (95% CI −0.17; −0.01) *P*=0.037, respectively. No other treatment effects were seen in models adjusted for nerve conduction velocity (Table S1). A borderline significant group difference was seen for MNSI score (Table 2). When models for vascular outcomes were adjusted for this possible confounder for patients with baseline measure of the confounder a treatment effect was seen as an increase in transcutaneous oxygen tension on the right foot and mean values for both feet at week 1 with an estimate of 1.08 mm Hg (95% CI 0.26; 1.89) (*P*=0.016) and 0.95 mm Hg (95% CI 0.2; 1.69) (*P*=0.019), respectively. No other treatment effects were seen in models adjusted for nerve conduction velocity (Table S2). We did not adjust tertiary neuropathy outcomes further for nerve conductance velocity or MNSI score as these analyses were adjusted for baseline measures of the specific measure of nerve function.

**Figure 3:**
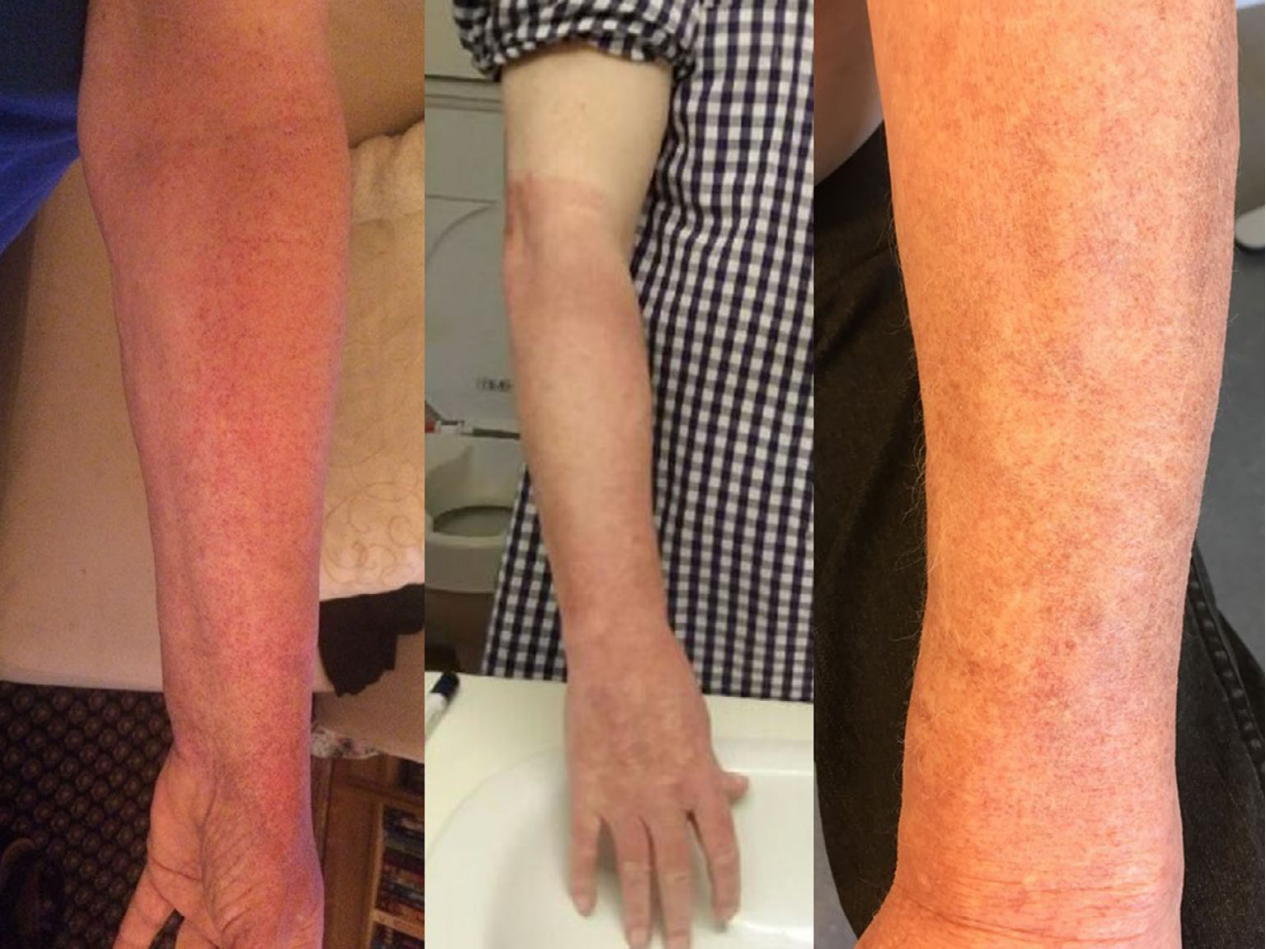
Adverse events. This figure shows pictures of the arm of all 3 patients experiencing adverse events.

Three patients experienced adverse events in the RIC treatment group. All patients experienced discomfort during cuff‐induced ischemia, and petechiae (microbleeds in the skin) appeared distal to the position of the cuff (Figure 3). Skin changes gradually disappeared within 1 week. No discomfort was experienced after treatment. All patients continued treatment on the other arm. One patient discontinued treatment because of reoccurrence of a similar adverse event on the other arm. None of the patients experienced persisting deficit, and no other adverse events were observed.

## Discussion

The results of the present study demonstrate that home‐based long‐term (12 weeks) treatment with repeated RIC is safe and feasible with remarkable compliance in a sham‐controlled setting. Despite adherence to treatment, RIC did not significantly improve tissue oxygenation or vascular or neuronal function in the lower extremities.

RIC treatment did not elicit significant improvements in microvascular perfusion of the feet assessed by transcutaneous tissue oxygen tension or macrovascular perfusion assessed by toe pressure and toe‐brachial index. It seems that results from previous studies in non‐diabetic cohorts demonstrating beneficial effects of RIC on endothelial function^10^ and microcirculation^11^ cannot be translated into an effect in diabetes mellitus patients. The paucity of improvements in vascular function in the present study may indicate that a vital parameter for quality of life in patients with diabetes mellitus and PAD—walking distance—may not be improved by RIC treatment as seen in non‐diabetic mellitus individuals without claudication treated for 6 weeks.^24^

RIC treatment has been shown to affect risk factors for increased arterial stiffness such^16^ as markers of inflammation.^17^ However, arterial stiffness assessed by PWV was not affected by RIC treatment in our study. Only one study has investigated the efficacy of RIC treatment on nerve function, showing that 6 weeks of RIC treatment improved autonomic function by increasing heart rate variability indices in nondiabetic individuals.^24^ However, we were not able to demonstrate any beneficial effects on measures of either autonomic or peripheral nerve function.

The lack of effect of RIC treatment on the outcome variables applied in this trial may be attributed to several factors. Treatment duration may be insufficient. Transcutaneous oxygen tension has been acutely improved in an acute study of RIC treatment;^23^ however, immediate effects may not extend beyond an acute reaction. However, prolonged RIC treatment for 6 and 8 weeks in healthy volunteers improved microvascular function assessed by cutaneous vascular conductance^11^ suggesting that RIC may modify microvascular function with a treatment duration shorter than applied in our study. Consequently, the treatment duration applied in the present study should be adequate to detect a response in microvascular function. Similarly, treatment duration should be sufficient to detect changes in PWV as several studies have demonstrated effect of drug intervention within 12 weeks of treatment.^25^ In addition, satin‐induced improvements in walking distance in patients with claudication has been seen after 6 to 12 weeks of treatment,^5^ suggesting that the vascular pathophysiology causing PAD may be affected within the duration of the present study.

We found not treatment effect on neuropathy measures. Neuropathy per se may attenuate treatment efficacy as peripheral neuropathy has been associated with reduced treatment effect.^6^ RIC stimulation of peripheral sensory nerves in the effector organ may be a prerequisite for initiation of a systemic response that is mediated by humoral or neural pathways.^6^ However, the impact of neural pathways remains unknown as cardio protection can be achieved by RIC in the denervated heart. Indeed, plasma from patients with diabetes mellitus and peripheral neuropathy has shown to have no cardioprotective attributes in experimental in vitro settings.^26^ Thus, the presence of neuropathy in the majority of our study population may explain the absent response to RIC. Sural nerve conductance velocity was marginally lower in the treatment group as the only objective measure of neuropathy. Additional adjustments for conductance velocity for vascular outcomes showed clinically negligible but statically significant reduction in transcutaneous oxygen tension in the right foot induced by RIC treatment. Similar effects were not seen for the other foot or means oxygen tension measures or other outcomes. When adjusting for MNSI‐count RIC treatment was associated with a small but statically significant improvement in transcutaneous oxygen tension at week 1. No effect of treatment was seen at other timepoints or other outcomes. We estimate that these findings are spurious, and that lack of treatment effect may be because of other factors than differences in nerve function in the 2 study arms.

The lack of treatment effect may be attributed to the extent of vascular damage present in the cohort. Despite having only moderately reduced toe pressure between 40 and 70 mm Hg, patients may have progressed beyond a point of reversibility by any treatment modality. In addition, the relative long diabetes mellitus duration of the study population may also reduce the effect of RIC, as duration of disease is associated with diminished treatment efficacy.^27^

Whether diabetes mellitus per se attenuates RIC efficacy is not clear.

Patients with PAD may experience transient ischemic episodes when physically active and therefore may be preconditioned and thus have no additional effect of RIC. However, intermittent claudication was not associated with reduced mortality in a study of patients with myocardial infarction,^28^ indicating that patients with PAD may not experience preconditioning as a consequence of their atherosclerotic disease.

The number of study participants may have been insufficient to show an effect of treatment. The power calculation was based on an expected effect in the primary outcomes <30% than seen in healthy volunteers. This could have been an overestimation of the effect in diabetes mellitus patients with PAD.

### Safety

Compliance to treatment protocol was remarkably high with compliance rates of 92.1% in the RIC group and 82.1% in the sham group. Three adverse events of transient skin petechiae distally to cuff placement were observed in the RIC group. All 3 patients were treated with acetylsalicylic acid. Adverse events were transient, and no permanent deficits were observed.

## Conclusion

Our study shows that in patients with type 2 diabetes mellitus and moderate peripheral artery disease 12‐week repeated remote ischemic conditioning treatment had no effect on tissue oxygenation, vascular or neuronal function despite remarkable compliance to treatment.

## Author Contributions

Hansen conceived and designed the research project, analyzed and interpreted the data, drafted the manuscript, and is the guarantor of this work; Jørgensen, Bøtker, Fleischer, and Rossing, analyzed and interpreted the data and made critical revision of the manuscript for key intellectual content.

## Sources of Funding

The project was funded by unrestricted research grant from The Augustinus Foundation (grant no. 15‐5043), Director Jacob Madsens Foundation (grant no. 5657‐2015/16), Steno Diabetes Center Research Foundation (grant no. 020216), The Toyota Foundation (grant no. KJ/BG‐8965 H.), Aase and Ejnar Danielsens Foundation (grant no. 10.001647), and Novo Nordisk Foundation (grant no. NNF15OC0017746).

## Disclosures

Fleischer J is co‐inventor of the medical device Vagus and hold stocks in Medicus Engineering. The remaining authors have no disclosures to report.

## Supporting information


**Table S1.** Effect of Trial Additionally Adjusted for Baseline Sural Nerve Conductance Velocity
**Table S2.** Effect of Trial Additionally Adjusted for Baseline Michigan Neuropathy Screening Instrument (MNSI) Score
