# Supplementary material for: Efficacy of Long‐Term Remote Ischemic Conditioning on Vascular and Neuronal Function in Type 2 Diabetes Patients With Peripheral Arterial Disease
Source: J Am Heart Assoc. 2019 Jun 25;8(13):e011779. doi: 10.1161/JAHA.118.011779 (PMC6662370; doi:10.1161/JAHA.118.011779)
Supplement: Supplementary file 1 — Table S1. Effect of Trial Additionally Adjusted for Baseline Sural Nerve Conductance Velocity Table S2. Effect of Trial Additionally Adjusted for Baseline Michigan Neuropathy Screening Instrument (MNSI) Score [file JAH3-8-e011779-s001.pdf]

SUPPLEMENTAL MATERIAL

Table S1. Effect of trial additionally adjusted for baseline sural nerve conductance velocity.

	Week 1			Week 4			Week 12		
	Active treatment	Placebo	Active vs. Placebo difference in change from baseline	Active treatment	Placebo	Active vs. Placebo difference in change from baseline	Active treatment	Placebo	Active vs. Placebo difference in change from baseline
Primary outcome									
Transcutaneous oxygen tension, right foot (mmHg)	50.8 (9.8)	46.7 (11.3)	0.24 (-0.59; 1.08) [0.571]	45.8 (14.4)	53 (10.8)	-0.36 (-0.59; -0.13) [0.003]	47.9 (13.4)	50.7 (8.1)	-0.09 (-0.17; -0.01) [0.037]
Transcutaneous oxygen tension, left foot (mmHg)	50.4 (11.2)	48.5 (12.4)	0.44 (-0.35; 1.22) [0.283]	50.5 (10.5)	54.2 (10.3)	-0.07 (-0.31; 0.16) [0.547]	51.2 (9.6)	51.5 (9.5)	-0.01 (-0.09; 0.07) [0.776]
Transcutaneous oxygen tension, mean (mmHg)	50.6 (9.2)	48.5 (12.2)	0.43 (-0.27; 1.13) [0.243]	48.1 (11.7)	54.3 (8.9)	-0.17 (-0.38; 0.03) [0.105]	49.5 (10)	51.8 (8.6)	-0.04 (-0.11; 0.03) [0.228]
Secondary outcomes									
Pulse wave velocity (m/s)	n/a	n/a	n/a	13.6 (3.9)	13.2 (3.4)	-0.15% (-0.49; 0.19) [0.402]	14.7 (3.7)	13.3 (3.3)	0.06% (-0.06; 0.19) [0.313]
Toe pressure, mean (mmHg)	n/a	n/a	n/a	n/a	n/a	n/a	76.6 (15.6)	69.8 (13.1)	-0.06 (-0.22; 0.10) [0.461]
Toe-brachial index, mean	n/a	n/a	n/a	n/a	n/a	n/a	0.5 (0.44;0.54)	0.5 (0.47;0.65)	-0.08% (-0.32; 0.15) [0.489]

Data are means (SD) or medians (IQR). Estimates of treatment effect are in % or absolute values (95% CI) [P values] for group difference. Models have been adjusted for baseline values of the given outcome and for mean sural nerve conduction velocity at baseline.

Table S2. Effect of trial additionally adjusted for baseline Michigan neuropathy screening instrument (MNSI) score.

	Week 1			Week 4			Week 12		
	Active treatment	Placebo	Active vs. Placebo difference in change from baseline	Active treatment	Placebo	Active vs. Placebo difference in change from baseline	Active treatment	Placebo	Active vs. Placebo difference in change from baseline
Primary outcome									
Transcutaneous oxygen tension, right foot (mmHg)	49.4 (10.9)	45.5 (8.1)	0.66 (-0.4; 1.72) [0.236]	17 45.8 (14.4)	9 51.3 (8.7)	-0.26 (-0.54; 0.02) [0.074]	16 47.8 (13.3)	9 51.3 (6.7)	-0.07 (-0.17; 0.02) [0.143]
Transcutaneous oxygen tension, left foot (mmHg)	49.2 (11.8)	47.2 (13.4)	1.08 (0.26; 1.89) [0.016]	17 50.9 (10.9)	11 52.5 (12)	0.08 (-0.19; 0.36) [0.55]	16 51.1 (9.5)	10 51.1 (9.6)	0.01 (-0.08; 0.1) [0.820]
Transcutaneous oxygen tension, mean (mmHg)	49.3 (10.1)	47.6 (11.8)	0.95 (0.2; 1.69) [0.019]	17 48.3 (11.9)	11 53 (9.9)	-0.04 (-0.29; 0.21) [0.748]	16 49.4 (9.9)	10 52.1 (7.9)	-0.02 (-0.1; 0.06) [0.609]
Secondary outcomes									
Pulse wave velocity (m/s)	n/a	n/a	n/a	13 13.7 (3.8)	8 13.6 (4.1)	-0.04% (-0.43; 0.35) [0.829]	15 (4.5)	13.4 (4)	0.09% (-0.04; 0.21) [0.183]
Toe pressure, mean (mmHg)	n/a	n/a	n/a	n/a	n/a	n/a	16 74.1 (14.9)	10 75.5 (16.1)	-0.02 (-0.2; 0.17) [0.848]
Toe-brachial index, mean	n/a	n/a	n/a	n/a	n/a	n/a	0.51 (0.44;0.58)	0.51 (0.43;0.65)	-0.05% (-0.31; 0.21) [0.685]

Data are means (SD) or medians (IQR). Estimates of treatment effect are in % or absolute values (95% CI) [P values] for group difference. Models have been adjusted for baseline values of the given outcome and for Michigan neuropathy screening instrument (MNSI) score at baseline.
